# N‐Heterocyclic Carbenes: Molecular Porters of Surface Mounted Ru‐Porphyrins

**DOI:** 10.1002/anie.202211877

**Published:** 2022-11-02

**Authors:** Peter Knecht, Dennis Meier, Joachim Reichert, David A. Duncan, Martin Schwarz, Johannes T. Küchle, Tien‐Lin Lee, Peter S. Deimel, Peter Feulner, Francesco Allegretti, Willi Auwärter, Guillaume Médard, Ari Paavo Seitsonen, Johannes V. Barth, Anthoula C. Papageorgiou

**Affiliations:** ^1^ Physics Department E20 Technical University of Munich James Franck Straße 1 85748 Garching Germany; ^2^ Diamond Light Source Harwell Science and Innovation Campus Didcot OX11 0QX UK; ^3^ Chair of Proteomics and Bioanalytics Technical University of Munich Emil Erlenmeyer Forum 5 85354 Freising Germany; ^4^ Département de Chimie École Normale Supérieure 24 rue Lhomond 75005 Paris France; ^5^ Université de recherche Paris-Sciences-et-Lettres Sorbonne Université Centre National de la Recherche Scientifique 75005 Paris France; ^6^ Department of Chemistry Laboratory of Physical Chemistry National and Kapodistrian University of Athens Panepistimiopolis 157 71 Athens Greece

**Keywords:** Metalloporphyrins, N-Heterocyclic Carbenes, Scanning Tunnelling Microscopy, Surface Chemistry, X-Ray Standing Waves

## Abstract

Ru‐porphyrins act as convenient pedestals for the assembly of N‐heterocyclic carbenes (NHCs) on solid surfaces. Upon deposition of a simple NHC ligand on a close packed Ru‐porphyrin monolayer, an extraordinary phenomenon can be observed: Ru‐porphyrin molecules are transferred from the silver surface to the next molecular layer. We have investigated the structural features and dynamics of this portering process and analysed the associated binding strengths and work function changes. A rearrangement of the molecular layer is induced by the NHC uptake: the NHC selective binding to the Ru causes the ejection of whole porphyrin molecules from the molecular layer on silver to the layer on top. This reorganisation can be reversed by thermally induced desorption of the NHC ligand. We anticipate that the understanding of such mass transport processes will have crucial implications for the functionalisation of surfaces with carbenes.

## Graphical abstract

Chemisorbing a prototypical N‐heterocyclic carbene (NHC) to a dense packed layer of metalloporphyrins pulls out complete metalloporphyrins into the second layer. This dynamic and reversible phenomenon was unraveled by structural, spectroscopic, desorption and theoretical investigations. The molecular rearrangement of the metallorganic layer is paralleled to NHCs pulling out single metal atoms from surfaces due to strong adsorption.

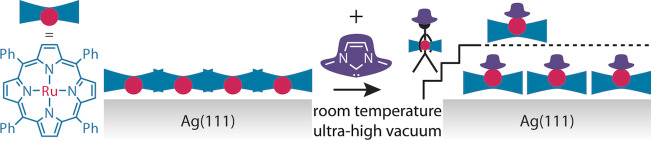

## Introduction

Single metal atoms on surfaces have attracted attention owing to the multitude of functional properties spanning from catalysis^[1]^ to magnetism^[2]^ they entail. Ligating such metal atoms can both stabilise and tune their physicochemical properties. Cyclic tetrapyrrole compounds, for example, can stabilise undercoordinated metal atoms on surfaces,^[3]^ whereas coordination of axial ligands has produced surface rotors^[4]^ and modified the atomic spin characteristics.^[5]^

N‐heterocyclic carbenes (NHCs) are an interesting class of ligands that offer robust bonding and promising properties in surface functionalisation.^[6]^ Several recent studies focused in unravelling their binding to well‐defined planar metal surfaces (Au,^[7]^ Cu,[^7h^, ^7i^, ^7j^, ^7k^, ^8^] Ag,[^7i^, ^7j^, ^7k^, ^8a^] and Pt)^[9]^ as well as (passivated) Si^[10]^ and oxidised Cu.^[11]^

**Figure 1:**
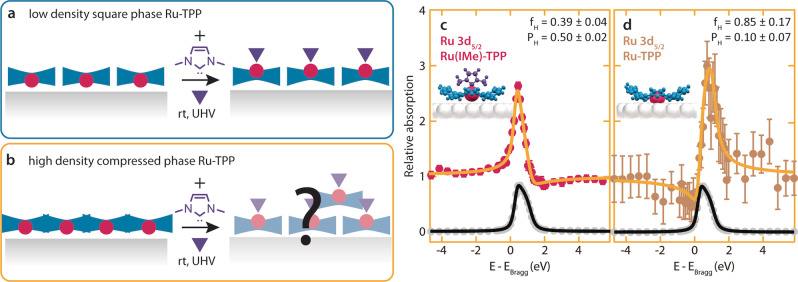
Adsorption height of Ru hosted in porphyrins on Ag(111) before and after NHC ligation. a,b) Schematic of the effects of IMe ligation on different layers of Ru‐TPP/Ag(111). In the square phase (a), the TPP self‐assembly is retained, the Ru centres are displaced further from the Ag interface. In the compressed phase (b), a uniform displacement of the Ru centres is not compatible with the NIXSW data. c,d) NIXSW absorption profiles following IMe ligated functionalisation of the compressed phase on Ag(111), of the Ru 3d_5/2_ core level from Ru(IMe)‐TPP (c) and Ru‐TPP (d). The yellow curves show the fits to the data, grey dots and black curves indicate the reflection of the silver substrate data points and fit, respectively. DFT models of Ru(IMe)‐TPP and Ru‐TPP on the Ag(111) surface are shown as insets.

Inspired by catalytically active porphyrin Ru‐NHC compounds,^[12]^ we recently used an NHC ligand to functionalise Ru centres in porphyrins on a silver surface.^[13]^ The modular construction of such interfaces worked remarkably well: the NHC was found to selectively ligate to the Ru centre of the pre‐assembled porphyrins on Ag(111) without disrupting their self‐assembly. Each Ru‐porphyrin molecule served as a pedestal for the precise surface orientation and packing of an NHC ligand. Diffraction‐based measurements showed that the NHC functionalised Ru atoms uniformly moved 0.9 Å away from the silver interface (illustrated in Figure 1a). This corresponds to a very strong structural surface *trans*‐effect,^[14]^ whereupon the surface is considered as a *trans* ligand to the Ru(NHC), whose bonding weakens.

Further investigations of this system illuminated an apparent paradox, which we report here: the assembly of the same NHC on the same Ru‐porphyrins, albeit in a different pre‐assembled form of higher density,^[15]^ on the same surface following the same procedure resulted in a non‐uniform adsorption height of the NHC ligated Ru (illustrated in Figure 1b). To elucidate this phenomenon observed by normal incidence X‐ray standing waves (NIXSW),^[16]^ we employed a structural determination methodology with real and reciprocal space imaging as well as thermal desorption experiments and work function measurements. Thereby we reveal an NHC induced dynamic rearrangement of the molecular film in two layers.

## Results and Discussion

We functionalised a saturated monolayer of Ru tetraphenyl porphyrin (Ru‐TPP, 5,10,15,20‐tetraphenyl‐21*H*,23*H*‐porphine ruthenium(II)) molecules on Ag(111), assembling in a compressed phase,^[15]^ with the NHC IMe (1,3‐dimethyl‐1,3‐dihydro‐2*H*‐imidazol‐2‐ylidene) at room temperature (rt, 300 K). The Ru 3d core level of Ru‐TPP/Ag(111) exhibits a characteristic energy shift by 1.2 eV after the IMe ligation (Figure S1). This allows us to perform NIXSW of the Ru in the different environments to deduce the respective adsorption height from the surface. Analysis of the Ru 3d_5/2_ component of the IMe ligated Ru (Figure 1c) in this saturated layer of Ru‐TPP on Ag(111) yields a coherent position, P_H_, of 0.50±0.02 and a coherent fraction, f_H_, of 0.39±0.04. The coherent position, which can be used to deduce the adsorption height of the IMe ligated Ru, increased significantly upon ligation, similarly to our recently reported results on the less densely packed square phase of Ru(IMe)‐TPP on Ag(111).[^13^, ^15^] However, the significant drop in the coherent fraction (from f_H_=0.88±0.09) is not compatible with a single adsorption height for the Ru centre and consequently not compatible with a uniform conformational change of each molecule as observed for the square phase ligation.[^13^, ^15^] To attribute the drop of the coherent fraction solely to IMe ligation and not to a potential variation in the adsorption height of the Ru‐TPP molecules on Ag(111) in the compressed phase, we simultaneously monitored the small fraction of uncapped Ru‐TPP molecules in the same layer (Figure S2,S3). The respective Ru 3d_5/2_ was found to have P_H_=0.10±0.07 and f_H_=0.85±0.17 (Figure 1d), in agreement with the values obtained for the compressed phase Ru‐TPP/Ag(111).^[15]^ From this, we can deduce that all the uncapped Ru atoms in this layer have an adsorption height of 2.59±0.17 Å, identical to both the compressed phase of Ru‐TPP/Ag(111) and the less dense square phase.^[15]^

This puzzling effect cannot stem from either the nature of the NHC‐Ru‐TPP ligation (we identified a well‐defined adsorption height for the square phase Ru(IMe)‐TPP) or from the change of the initial position of Ru (in both self‐assembled structures Ru was found to have the same well‐defined adsorption height). To understand this phenomenon, we investigated the self‐assembly with scanning tunnelling microscopy (STM) and low‐energy electron diffraction (LEED). While STM offers a unique way of visualising the self‐assembly in real space, thus being sensitive to deviations from regularity, LEED facilitates the easier recognition of even minute changes in the periodic arrangement.

**Figure 2:**
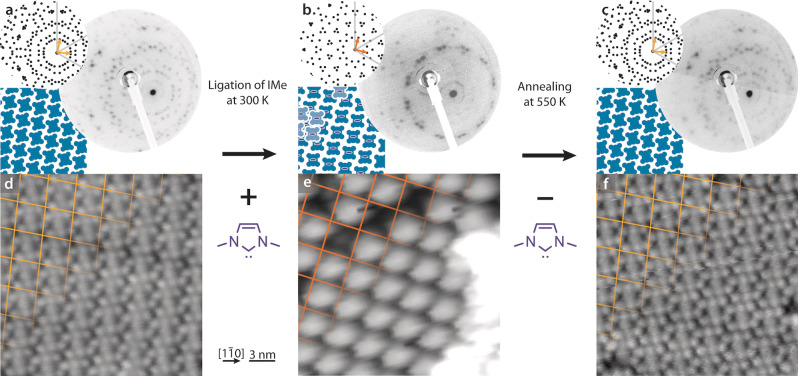
Porter effect of IMe monitored by LEED and STM. a,d) Ru‐TPP on Ag(111) self‐assembled in the compressed phase. b,e) The same sample following deposition of IMe shows a diffraction pattern matching the square phase Ru(IMe)‐TPP, the STM image confirms the phase change and indicates an accumulation of molecules in a second layer. c,f) After annealing the surface imaged in b/e to 550 K, the compressed phase LEED pattern observed in a is restored. All LEED images were taken at 300 K, the simulated diffraction pattern of each phase is plotted next to the LEED image by black dots, the high symmetry axes of the substrate are indicated in grey, the unit cell vectors of a single domain are shown. In the middle, a schematic, top view representation of the corresponding real‐space arrangements of the Ru‐TPP (blue shapes) and IMe ligands (purple ellipses) are shown. STM parameters: −1.3 V, 50 pA, 300 K (d), 2.1 V, 60 pA, 140 K (e), −1.8 V, 70 pA, 230 K (f). The overlaid grids highlight the self‐assembly.

The LEED pattern of the compressed phase before IMe ligation is shown in Figure 2a, with the primitive vectors of one of the six equivalent unit cells highlighted. The inset shows the characteristic simulated pattern based on an overlayer matrix of $\left(\begin{array}{cc} 4 & 5\\ 3 & -13/6 \end{array}\right)$ ^[15]^ for comparison with the experimental observation. The corresponding overview STM image (Figure 2d) shows the Ag(111) surface covered with Ru‐TPP, where each protrusion signifies a single Ru‐TPP molecule. A grid matching the expected periodicity from LEED is superposed on the top left corner. After depositing IMe at rt on this monolayer, we observe marked differences (Figure 2b). The LEED pattern undergoes a transformation, which can be identified as the pattern of the sparser square phase. Based on the unit cell identified by LEED, the DFT optimised structure reveals a uniform distribution of the Ru(IMe)‐TPP molecules (Figure S4) described by the overlayer matrix $\left(\begin{array}{cc} 11/2 & 3/2\\ 3/2 & 11/2 \end{array}\right)$ . The transformation of the contact layer to the square phase can be confirmed in the real space imaging (Figure 2e), which is highlighted on the top left corner by the grid marking the molecular density. Here the vast majority of Ru‐TPP molecules are ligated to IMe and can be identified as the brighter protrusions of the same plane.^[13]^

This reduced density indicates that a fraction of ≈20 % of the initially adsorbed Ru‐TPP molecules is missing from the Ag(111) interface. Strikingly, the phase transformation is reversible: after annealing the IMe ligated Ru‐TPP to a temperature, which entails desorption of IMe (cf. TPD investigation below), the LEED and STM data show that the initial compressed phase is restored (Figure 2c,f and Figure S5). Additionally, the XPS intensities related to the Ru‐TPP remain essentially unaffected during this transformation (Figure S1, S6). Desorption of the missing Ru(IMe)‐TPP molecules can therefore be ruled out. For this transformation, manifestly, the NHCs (depicted schematically as purple ellipses in Figure 2b) act as molecular porters that carry ≈20 % of the Ru‐TPP molecules (depicted in blue) from the silver contact layer to a second layer on top. It is expected for Ru(NHC)‐TPP to diffuse as a single entity instead of NHC hoping across the distant Ru binding sites.

Such relocation is also evident in the STM images (Figure 2e, S7, S8). Small molecular adlayers on top of the first layer can be identified by imaging at 140 K, where protrusions of similar appearance to the regular underlying structure can be discriminated (Figure S8). Line profiles across these adlayers suggest that they are not related to a local island formation of the silver surface underneath, as the related step height does not match the atomic step height of Ag(111). The porphyrin molecules of the second layer are frequently found self‐assembling in islands with densities of up to 0.49 molecules nm^−2^. They have predominantly equal apparent heights and registry, occupying sites centred in between the contact layer porphyrins (see inset of Figure S8b). The adlayers cannot be visualised by STM investigations at rt, presumably due to increased mobility and prevention of possible porphyrin‐porphyrin interactions upon NHC ligation. Indeed, in the absence of the NHC ligand, stacking of Zn‐TPP second layer molecules was observed at rt,^[17]^ indicating stronger interactions. In contrast, when the central moiety of the tetrapyrrole macrocycle is strongly bonding to the substrate^[18]^ or it is out of the macrocycle plane,^[19]^ a lateral offset is observed in the registry of the second layer as in the case here. STM investigations of our system at rt revealed surfaces wetted only by a uniform single layer of porphyrins.

The second layer created by the porter effect contributes to the NIXSW with a different adsorption height for both the C 1s and the Ru 3d_5/2_. This has a more pronounced effect on the Ru 3d_5/2_ spectra (Figure 1c). Indeed, a small contribution of the second layer Ru centres is sufficient to lower the coherent fraction significantly, owing to the interference of the emitted electrons from the two different adsorption heights. The coherent position of 0.50±0.02 (Figure 1c) would correspond to an adsorption height of 3.53±0.05 Å in the case of a single adsorption site. However, with two different adsorption heights, it is not possible to determine the two different adsorption heights precisely. The measured coherent position and the transformation to the square phase self‐assembly suggest that the adsorption height of the Ru atoms of the molecules at the Ag(111) interface is comparable to the 3.48±0.10 Å measured for square phase Ru(NHC)‐TPP on Ag(111),^[13]^ with a respective structural *trans*‐effect resulting in a displacement of approximately 0.9 Å away from the surface.

While the portering also influences the absorption profiles of the C 1s core levels (Figure S9), the effect is less dramatic due to the non‐planarity of the porphyrin macrocycle. The coherent position increases significantly from 0.29±0.02^[15]^ to 0.52±0.01 upon ligation, indicating that not only the Ru centre is experiencing the *trans*‐effect, but that the whole porphyrin macrocycle changes its shape, akin to the submonolayer coverages of Ru(NHC)‐TPP on Ag(111).^[13]^ The coherent fraction is already very low for the pristine Ru‐TPP layer (0.16±0.02), signalling that the carbon atoms reside at multiple different adsorption heights, and reduces further (0.13±0.01) after IMe ligation, supporting the transfer of the complete molecule to the second layer.

The mass transport induced by the portering of entire molecules is reminiscent of the NHC induced removal of single metal atoms from the coinage metal surfaces[^7c^, ^7g^, ^7k^] and their surface transport.[^7f^, ^7h^] However, to the best of our knowledge, this is the first report of molecules being carried from the metal surface to the next layer. We attribute the physical origin of this phenomenon to the relative adsorption strength of Ru‐TPP and Ru(IMe)‐TPP on Ag(111). Indeed, DFT calculations reveal that IMe weakens the bond of Ru‐TPP to the Ag surface (detailed energies in Figure S10). This can be described by the *trans‐*effect: ligation of the IMe ligand *trans* to the Ag(111) surface results in a weakening of the Ru−Ag bond. As the formation of the compressed phase is the result of the strong bonding between the Ru‐TPP and the Ag(111),^[15]^ within this phase we see the ultimate demonstration of the *trans*‐effect: breaking of the Ru−Ag bond. It is further conceivable that the porphyrin's structural rearrangement upon IMe ligation influences steric contributions of the porter effect, however it is noted that the surface footprint is not affected by the ligation (see Figure S10 for comparison).

The *trans*‐effect is documented upon ligation of several inorganic molecules on metalloporphyrins and phthalocyanines. In particular, a substantial structural *trans*‐effect is observed on the Ru‐TPP/Ag(111) upon CO ligation: the Ru atoms are displaced 0.6 Å further away from the Ag interface, while they remain very close to their initial adsorption sites.^[14e]^ In contrast, the strong registry on Ag(111) hollow sites is not found after IMe ligation in the DFT optimised geometries (Figure S4). And, importantly, it is notable that such a porter effect cannot be observed on the corresponding LEED experiment when CO replaces IMe (Figure S11), in an experiment albeit performed at a 100 K lower temperature required by the lower desorption energy of CO.^[14e]^

**Figure 3:**
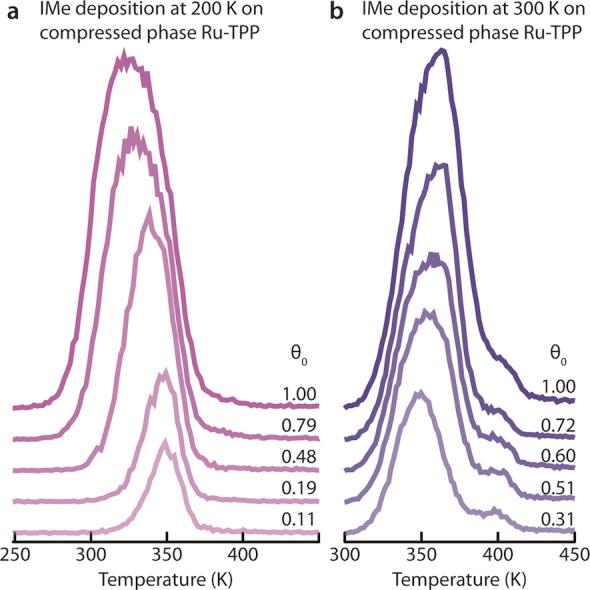
TPD spectra of IMe on Ru‐TPP (*m*/*z*=96, corresponding to the parent ion of IMe). Each graph shows spectra for different initial coverages of IMe, θ_0_, up to a saturation of all Ru‐TPP molecules (θ_0_=1). The spectra are offset along the vertical axes for clarity. a) IMe (T_sample_=200 K during sublimation) on a compressed layer Ru‐TPP. b) IMe (T_sample_=300 K during sublimation) on a compressed layer Ru‐TPP.

To investigate thermal effects on the observed phase transformation, IMe was also deposited on the compressed Ru‐TPP phase at 200 K. By monitoring the binding energy of the Ru 3d_5/2_ region in XPS, we could confirm the formation of a Ru(IMe)‐TPP layer, but no difference was identified in the respective LEED pattern (Figure S12). Thus, we can conclude that the porter effect is thermally assisted and hitherto particular to IMe. We therefore investigated the dynamics of IMe on the Ru‐TPP compressed phase on Ag(111) with TPD experiments. Figure 3a shows the TPD spectra after the deposition of IMe at 200 K on Ru‐TPP in the compressed phase. While at low initial IMe coverages (θ_0_) the apex of the desorption peak is located at ≈350 K, it is shifted towards lower temperature with increasing coverages of IMe. Gratifyingly, with the assumption of repulsive interactions between adsorbates, this effect could be modelled with an equation featuring decreasing desorption energy for increasing coverage (the respective analysis is outlined in the Supporting Information). The best fitting results (Figure S13) were obtained with a frequency factor $\nu =\;1.04\times 10^{15}\;s^{-1}$ , which falls into the range of common frequency factors reported for desorption of organic molecules,^[20]^ and a desorption energy of $E_{des}=\left(1.08-0.12\cdot \theta \right)\;eV$ . From our analysis, the determined desorption energy of IMe from Ru‐TPP/Ag(111) ranges between 0.96 eV (all Ru centres covered) and 1.08 eV (zero coverage limit). These energies are consistent with the earlier reported data^[13]^ by computation with the same frequency factor $\nu =1.04\times 10^{15}\;s^{-1}$ .

**Figure 4:**
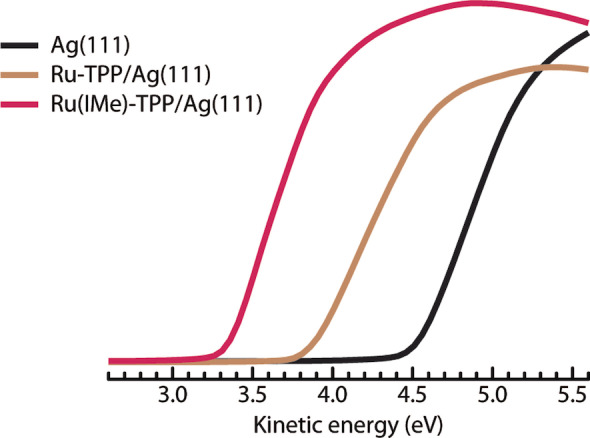
Work function changes in the Ru‐TPP and Ru(IMe)‐TPP functionalised Ag(111). The XPS secondary electron cutoff for the bare Ag(111) surface (black), Ru‐TPP/Ag(111) in the compressed phase (brown) and Ru(IMe)‐TPP/Ag(111) (red).

We propose that the repulsion is caused in first approximation by dipole‐dipole interactions^[21]^ between adjacent Ru(IMe)‐TPP molecules. Since substantial molecular dipoles are also evident in DFT simulations for a range of NHCs adsorbed directly on a metal surface and also have been observed in work function measurements,[^7l^, ^22^] it would be expected for such interactions to influence the thermal stability of NHC self‐assembled monolayers. The accumulation of surface dipoles associated with the NHC adsorption also seem to play a role in the expression of the porter phenomenon: an STM investigation of the effect of the IMe coverage (Figure S14) revealed that the related adlayers form only after a high coverage is reached (θ=0.72) and that the effect is extended for higher IMe coverages (evidenced by complete transformation to the square phase, Figure S8). Importantly, the desorption energy reduction of 0.12 eV could be reproduced by a dipole moment of ≈6 D (see Supporting Information for calculation), in very good agreement with DFT, from which we estimate this dipole moment to be 5.3 D. Such dipoles result in a reduction of the work function upon IMe adsorption. This was quantified as 0.52 eV by the XPS secondary electron cutoff, in line with DFT calculations (Figure 4 and Table 1).

**Table 1:** Work function (Φ) values with respect to the bare Ag(111) surface, measured by XPS and calculated with DFT. The experimental data were acquired on a surface with 0.58  molecules nm^−2^. DFT calculations considered a surface with 0.49 molecules nm^−2^.

	XPS	DFT
Φ_Ag(111)_ – Φ_Ru‐TPP/Ag(111)_	0.67±0.02 eV	0.65 eV
Φ_Ag(111)_ – Φ_Ru(IMe)‐TPP/Ag(111)_	1.19±0.02 eV	1.42 eV

With the onset of IMe desorption already at ≈270 K for high coverages, the ligation of the compressed phase should not be stable at 300 K. Surprisingly, complete NHC functionalisation of the Ru centres was nonetheless achieved at 300 K. TPD spectra of IMe deposited at 300 K are shown in Figure 3b. While at low IMe coverages, the main peaks are comparable to the desorption spectra shown in Figure 3a, there is an additional component at ≈400 K. This component is not observed in either the TPD of the compressed phase after exposure to IMe at 200 K, nor the square phase after exposure to IMe at 300 K.^[13]^ It is therefore reasonable to assign this component to IMe desorption from the transported Ru‐TPP molecules and to the reintegration of those Ru‐TPP molecules back into the compressed phase. This is in good accord with the stronger binding of ligands to the Ru‐TPP adlayer vs. to Ru‐TPP in direct contact with Ag(111) observed experimentally for CO.^[14e]^ Indeed, DFT calculations find an energy difference of 1.05 eV between the bond strength of IMe to the isolated Ru‐TPP molecule vs. to Ru‐TPP adsorbed on Ag(111).

## Conclusion

We have discovered a fascinating, dynamic interface rearrangement based on the NHC ligand acting as a molecular porter for Ru‐TPP molecules on Ag(111). Ru(NHC)‐TPP complexes were lifted to a second layer to allow a phase transformation of the first layer from a compressed phase into the less densely packed square phase. Since the Ru‐TPP submonolayer porphyrin phase is unaffected in periodicity by the NHC ligation,^[13]^ the NHC porter phenomenon is anticipated only for metalloporphyrins forming condensed monolayers of higher densities. This mass transport is thermally activated and specific for the NHC ligand, which leads to weakened bonding of the ruthenium atom to the silver surface. Remarkably, this reorganisation is thermally reversible by annealing‐promoted removal of the NHC ligands. To the best of our knowledge, this is the first example of a reversible ejection of complete molecules from a monolayer.

Detailed analysis of the thermal desorption signals the effect of repulsive dipole interactions in the binding energy of NHCs on surfaces, and similar modelling is expected to be applicable in the characterisation of densely packed NHCs. We anticipate that harnessing these dynamic events is at hand and will serve the engineering of atomically precise NHC‐containing complex interfaces.

## Conflict of interest

The authors declare no conflict of interest.

1

## Supporting information

As a service to our authors and readers, this journal provides supporting information supplied by the authors. Such materials are peer reviewed and may be re‐organized for online delivery, but are not copy‐edited or typeset. Technical support issues arising from supporting information (other than missing files) should be addressed to the authors.

Supporting Information

## Data Availability

The data that support the findings of this study are available from the corresponding author upon reasonable request.
