# Supplementary material for: N‐Heterocyclic Carbenes: Molecular Porters of Surface Mounted Ru‐Porphyrins
Source: Angew Chem Int Ed Engl. 2022 Nov 2;61(49):e202211877. doi: 10.1002/anie.202211877 (PMC10092334; doi:10.1002/anie.202211877)
Supplement: Supplementary file 1 — Supporting Information [file ANIE-61-0-s001.pdf]

Angewandte Chemie
Eine Zeitschrift der Gesellschaft Deutscher Chemiker

Supporting Information

N-Heterocyclic Carbenes: Molecular Porters of Surface Mounted Ru-Porphyrins

*P. Knecht, D. Meier, J. Reichert, D. A. Duncan, M. Schwarz, J. T. Küchle, T.-L. Lee, P. S. Deimel, P. Feulner, F. Allegretti, W. Auwärter, G. Médard, A. P. Seitsonen, J. V. Barth, A. C. Papageorgiou**

Contents

Methods . . . S-2
Sample preparation . . . S-2
STM . . . S-2
XPS . . . S-2
Work function determination . . . S-2
LEED . . . S-3
TPD . . . S-3
TPD analysis . . . S-4
NIXSW . . . S-4
DFT . . . S-4
Dipole strength estimation . . . S-5
Additional Experimental Data . . . S-5
Figure S1: XPS of Ru(IMe)-TPP & Ru-TPP . . . S-6
Figure S2: Sum of Ru $3d_{5/2}$ spectra for NIXSW . . . S-7
Figure S3: Fits of single Ru $3d_{5/2}$ spectra for NIXSW . . . S-8
Figure S4: DFT model of Ru(IMe)-TPP on Ag(111) . . . S-9
Figure S5: LEED of Ru(IMe)-TPP . . . S-9
Figure S6: XPS of Ru-TPP . . . S-10
Figure S7: STM images of Ru-TPP and Ru(IMe)-TPP . . . S-11
Figure S8: STM images and line profiles of Ru(IMe)-TPP and Ag(111) . . . S-12
Figure S9: NIXSW of the C 1s signal in Ru(IMe)-TPP . . . S-13
Figure S10: DFT model Ru-TPP and Ru(IMe)-TPP on Ag(111) . . . S-13
Figure S11: LEED, STM and XPS of CO ligation on Ru-TPP at 200 K . . . S-14
Figure S12: LEED (20 eV) and XPS of IMe ligation on Ru-TPP at 200 K . . . S-14
Figure S13: Fitted TPD spectra . . . S-15
Figure S14: STM images of Ru-TPP with different dosages of IMe . . . S-16
Figure S15: LEED of Ru-TPP . . . S-17

Methods

Sample preparation

Three different ultra-high vacuum setups were used to perform the measurements. In all chambers, similar procedures were employed to prepare the samples. The Ag(111) single crystal went through several cycles of Ar^+ or Ne^+ sputtering and subsequent annealing to 725 K.

Ru-TPP was deposited onto the Ag(111) crystal, which was held at 300 K, by organic molecular beam epitaxy (OMBE) of Ru(CO)-TPP (Sigma Aldrich, 80 % dye content) at 550 K to 625 K. It has been shown, that clean Ru-TPP monolayers are obtained as the CO detaches from the Ru-TPP in this process and the purity of the compound can be enhanced by thorough outgassing.[1] The deposition rates were 0.3 $\frac{\text{molecules}}{\text{nm}^2\,\text{h}}$ to 2.0 $\frac{\text{molecules}}{\text{nm}^2\,\text{h}}$, depending on the temperature and chamber geometry. Monolayers of Ru-TPP self-assembled in the compressed phase were obtained by multilayer desorption at 550 K.

[1] Knecht, P., Ryan, P. T. P., Duncan, D. A., Jiang, L., Reichert, J., Deimel, P. S., Haag, F., Küchle, J., Allegretti, F., Schwarz, M., Garnica, M., Auwärter, W., Seitsonen, A. P., Barth, J. V. & Papageorgiou, A. C., Tunable interface of ruthenium porphyrins and silver. *J. Phys. Chem. C* **125**, 3215-3224 (2021).

Annealing (sub)monolayer coverages of Ru-TPP on Ag(111) at 550 K does not influence the self-assembly phase (Figure S15).

The masked IMe (1,3-dimethyl-1*H*-imidazol-3-ium-2-carboxylate,IMe-CO_2) was synthesized according to a reported procedure.[2] The free IMe was similarly deposited via OMBE by heating the masked IMe to temperatures of 360 K to 390 K. Deposition rates of up to 10 $\frac{\text{molecules}}{\text{nm}^2\,\text{h}}$ were observed on Ru-TPP monolayer samples held at 300 K, whereas at 200 K the rates were $\sim$5 times higher.

STM

An Aarhus-type STM (SPECS GmbH) was used. The STM was operated in constant current mode with a chemically etched W tip, the tunnelling bias was applied to the sample.

XPS

XP spectra were acquired with a Mg Kα source and a SPECS Phoibos 100 CCD hemispherical analyser in normal emission geometry. The energy scale was calibrated using the Ag $3d_{5/2}$ peak at a binding energy of 368.27 eV.

Work function determination

The work function was determined by the detection of the secondary electron cutoff by using the following relationship:

$\Phi = h\nu - |E_{\text{Fermi level}} - E_{\text{secondary electron cutoff}}|$

where Φ is the work function, $h\nu$ is the excitation photon energy, $E_{\text{Fermi level}}$ is the energy of the Fermi edge and $E_{\text{secondary electron cutoff}}$ is the energy of the secondary electron cutoff. For the measurements of the secondary electron cutoff, the sample was set to a potential of -20 V with respect to the electron analyser. The electrons were excited with Al Kα ($h\nu = 1486.61$ eV) source and detected by a SPECS Phoibos 100 CCD hemispherical analyser in normal emission geometry. The energy scale was calibrated by the Ag $3d_{5/2}$ peak at a binding energy of 368.27 eV. The data collected for the work function determination are presented in the following figure against a calibrated energy scale. The secondary electron cutoff (marked by a dashed line for the data corresponding to the clean Ag(111) surface) in kinetic energy scale amounts to the work function ($\Phi_{\text{Ag(111)}}$). The data presented in manuscript Figure 4 are a zoom in the secondary electron cutoff region vs. kinetic energy.

[2] Jiang, L., Zhang, B., Médard, G., Seitsonen, A. P., Haag, F., Allegretti, F., Reichert, J., Kuster, B., Barth, J. V. & Papageorgiou, A. C., N-Heterocyclic carbenes on close-packed coinage metal surfaces: bis-carbene metal adatom bonding scheme of monolayer films on Au, Ag and Cu, *Chem. Sci.* **8**, 8301-8308 (2017).

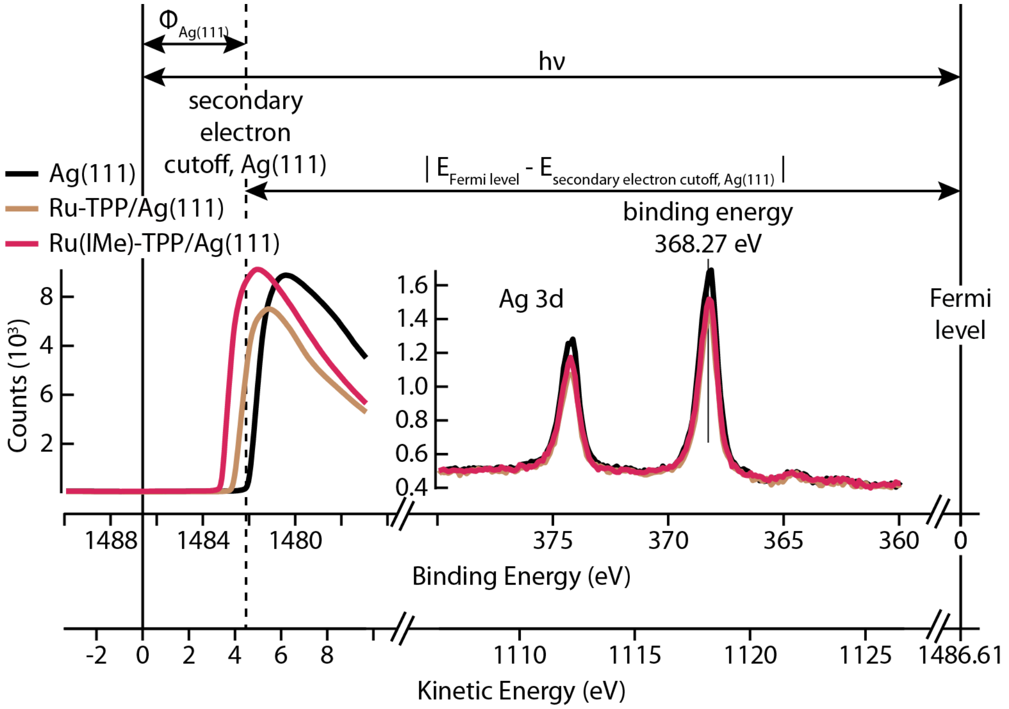

LEED

LEED measurements were performed with a BDL800IR-LMX-ISH by OCI Vacuum Microengineering Inc. All images were acquired with an electron energy E = 20 eV. Subtle changes in spot size of the same LEED pattern are associated with slightly worse molecular ordering or presence of spurious defects, e.g., reduced extension of the crystalline domain size.

TPD

Measurements were performed using a quadrupole mass spectrometer behind a copper cap cooled with LN_2,[3,4] with the sample located at a distance of $\sim$ 1 mm from the opening on the apex of the copper cap. All here reported spectra show the parent ion of IMe with m/z = 96. The heating rate for all spectra was set to $\beta = 5\,\mathrm{K\,s^{-1}}$.

TPD Analysis

Assuming that the desorption curves can be described by the Polanyi-Wigner equation, the peak temperature with increasing initial coverage θ_0 can be modelled by a coverage dependent desorption energy E_{des}. The inclusion of a decreasing desorption energy at increasing coverage θ can be used as a model

[3]Feulner, P. & Menzel, D., Simple ways to improve 'Flash Desorption' Measurements from Single Crystal Surfaces., J. Vac. Sci. Technol., 1980, 17 (2), 662-663.

[4]Frigo, S. P., Feulner, P., Kassühlke, B., Keller, C. & Menzel, D., Observation of neutral atomic fragments for specific $1s$ core excitations of an adsorbed molecule, *Phys. Rev. Lett.* **80**, 2813-2816 (1998).

for repulsive interactions.[5,6] We employ a linear decrease of the desorption energy with coverage, leading to the following equation:

$$\frac{d\theta}{dT} = \frac{v}{\beta} \cdot \theta \cdot \exp\left(-\frac{E_{des} - E_{\theta} \cdot \theta}{k_B T}\right) \tag{1}$$

This model replicates the shape of the TPD spectra as shown in Figure S13.

NIXSW

X-ray standing wave profiles were acquired at the I09 beam line at the Diamond Light Source.[7] All measurements were acquired with the sample held at ~200 K, using a Scienta EW4000 HAXPES analyser that was mounted perpendicular to the incident X-rays in the horizontal plane of the photon linear polarisation. Measurements for the (111) Bragg reflection with Bragg diffraction planes parallel to the surface were performed at a normal incidence Bragg energy of $h\nu = 2.63\,\text{keV}$. All measurements were repeated multiple times at different spots of the sample, where at each spot the reflectivity curve was measured to allow a precise energy alignment of the individual NIXSW measurements and to ensure the crystalline quality of the Ag(111). Monitoring of potential beam damage was performed by recording XP spectra of the C 1s and Ru 3d region before and after each NIXSW measurement.

DFT

The DFT geometry optimisation was carried out via the Quantum ESPRESSO[8] package. Within the vdW-DF2-B86r approximation[9] in the exchange-correlation term, five layers of the Ag(111) substrate were considered, the two lower layers fixed at their bulk-terminated positions. An optimized lattice constant of 4.1325 Å, 2×2 k points, Fermi–Dirac smearing of occupation numbers with a 50 meV broadening, projector augmented wave (PAW)[10] data sets for the pseudization of the core electrons, surface–dipole corrections, and cutoff energies of 60 Ry for the wave functions and 350 Ry for the electron density were applied. The unit cell included two molecules, as derived for square phase Ru-TPP by STM and LEED.[1]

Dipole strength estimation

The reduction of the adsorption energy due to dipole interactions is estimated in a simple model on the basis of Coulomb-interactions between dipoles. The potential energy between two point charges q_1 and q_2 with a separation r is given by

$$E_{pot} = \frac{1}{4\pi\epsilon_0}\frac{q_1 q_2}{r} \tag{2}$$

[5] Albano, E. V. Thermal desorption mass spectrometry of alkali metal atoms from transition metal surfaces. The influence of coadsorbed oxygen. *J. Chem. Phys.* **85**, 1044 (1986).

[6] Albano, E. V. A model for the work function change caused by coadsorption. *Appl. Surf. Sci.* **14**, 183 (1983).

[7] Lee, T.-L. & Duncan, D. A., A two-color beamline for electron spectroscopies at Diamond Light Source. *Synchrotron Radiat. News* **31**, 16-22 (2018).

[8] Giannozzi, P., Baroni, S., Bonini, N., Calandra, M., Car, R., Cavazzoni, C., Ceresoli, D., Chiarotti, G. L., Cococcioni, M., Dabo, I., Dal Corso, A., de Gironcoli, S., Fabris, S., Fratesi, G., Gebauer, R., Gerstmann, U., Gougoussis, C., Kokalj, A., Lazzeri, M., Martin-Samos, L., Marzari, N., Mauri, F., Mazzarello, R., Paolini, S., Pasquarello, A., Paulatto, L., Sbraccia, C., Scandolo, S., Sclauzero, G., Seitsonen, A. P., Smogunov, A., Umari, P. & Wentzcovitch, R. M., Quantum ESPRESSO: a Modular and Open-Source Software Project for Quantum Simulations of Materials. *J. Phys.: Condens. Matter* **21**, 395502 (2009).

[9] Hamada, I., Van der Waals density functional made accurate. *Phys. Rev. B* **89**, 121103 (2014); Lee, K., Murray, É. D., Kong, L., Lundqvist, B. I. & Langreth, D. C., Higher-accuracy van der Waals density functional. *Phys. Rev. B* **82**, 081101 (2010).

[10] Blöchl, P. E., Jepsen, O. & Andersen, O. K., Improved Tetrahedron Method for Brillouin-Zone Integrations. *Phys. Rev. B* **49**, 16223-16233 (1994).

To extend this to dipoles, the two charges of each dipole, separated by distance l, have to be considered. A superposition of energy contributions from surrounding dipoles, assuming a full coverage of IMe, leads to the following equation.

$$E_{Dip} = \frac{q^2}{4\pi\epsilon_0} \sum_{i=1}^{N} \left(\frac{2}{d_i} - \frac{2}{\sqrt{d_i^2 + l^2}} \right) \tag{3}$$

Including now as a last step image dipoles, induced by the conductive Ag(111) substrate, we obtain

$$E_{Dip} = \frac{q^2}{4\pi\epsilon_0} \sum_{i=1}^{N} \left(\frac{2}{d_i} - \frac{2}{\sqrt{d_i^2 + l^2}} + \frac{2}{\sqrt{d_i^2 + (l+s)^2}} - \frac{1}{\sqrt{d_i^2 + s^2}} - \frac{1}{\sqrt{d_i^2 + (2l+s)^2}} \right) \tag{4}$$

Here s is the distance between a dipole and its image dipole. With estimates for the required charges and lengths (q = 0.3 e, obtained from DFT as charge on the IMe; l = 4 Å as an estimation for the separation of IMe and the TPP macrocycle; s = 6 Å as an approximation of two times the Ag(111)/Ru-TPP separation), which results in a dipole moment of $p = q \cdot l = 6$ D, $E_{Dip} = 0.12$ eV is obtained, assuming a square assembly of the Ru(IMe)-TPP molecules with a spacing of 1.3 nm, determined for the compressed phase. The energy difference between calculations with total numbers N of surrounding dipoles of 10000 and 40000 was less than 1 %.

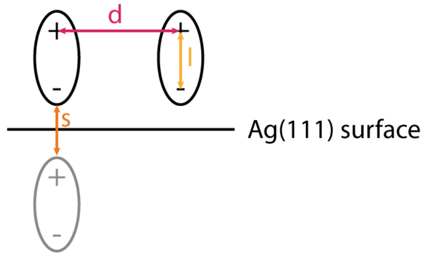

Scheme of the dipole-dipole interaction. The two dipoles (black ellipses) are separated by distance d, the charges in each dipole by l, the distance between dipole and image dipole (grey) is s.

Additional Experimental Data

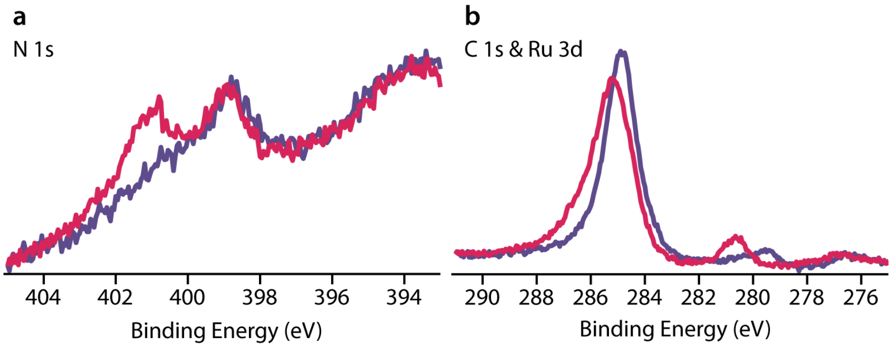

Figure S1: XP spectra of pristine Ru-TPP in the compressed phase (purple) and the same surface following IMe ligation at room temperature (red). (a) N 1s spectra. Upon IMe ligation, a second peak related to IMe (E_B = 401.1 eV) can be observed, while the first component related to Ru-TPP (E_B = 399.0 eV) does not change. (b) C 1s & Ru 3d spectra. Upon ligation, the shape of the carbon peak changes and the Ru $3d_{5/2}$ peak shifts from 279.4 eV (Ru-TPP) to 280.6 eV (Ru(IMe)-TPP).[11] The shift in energy is consistent with a decoupling of the Ru-TPP from the Ag(111) surface by the introduction of an axial ligand, firstly reported for Co-TPP with NO.[12] For the particular metalloporphyrin investigated here (Ru-TPP) on Ag(111), a shift to 281.8 eV is found after ligation of CO.[3] The slightly lower energy shift induced by IMe can be attributed to the electron donating character of the ligand, evidenced further in the DFT and TPD analyses as well as the work function measurements, presented in the manuscript. Additionally, an increase of the Ru $3d_{5/2}$ signal can be observed. This is similar to the XPS changes observed after CO axial ligation on the Ru atoms of Ru-TPP/Ag(111)[13] and is tentatively attributed to the change of the Ru environment, promoting forward scattering of the emitted photoelectrons. This effect is enhanced at high kinetic energy, due to the specific angular dependence of the atomic scattering factor for heavy metals.[14] Another probable, concomitant cause is the change of the coordination of the Ru upon ligation, which alters the crystal splitting of its d-band. Combined with decoupling the Ru from the metallic substrate (and thus the substrate's delocalized electrons), it could substantially alter the loss structure of the core level, resulting in the apparent increase in intensity in those spectra. The C 1s XPS signal changes in shape, and its total area increases by 5%. A shape change is expected due to the contribution from the C 1s of the IMe ligand at slightly higher binding energies,[2] resulting in the shoulder visible to the left of the main peak as well as the Ru $3d_{3/2}$ contribution shifting from 283.6 eV to 284.8 eV. We further note that the rearrangement of the surface might cause shadowing, which will affect the related peak intensities. No noticeable screening effect is expected for Ru(IMe)-TPP on Ag(111) based on earlier studies of Ru(CO)-TPP on the same surface.[13]

[11] Knecht, P., Zhang, B., Reichert, J., Duncan, D. A., Schwarz, M., Haag, F., Ryan, P. T. P., Lee, T.-L., Deimel, P. S., Feulner, P., Allegretti, F., Auwärter, W., Médard, G., Seitsonen, A. P., Barth, J. V. & Papageorgiou, A. C. Assembly and manipulation of a prototypical N-heterocyclic carbene with a metalloporphyrin pedestal on a solid surface. *J. Am. Chem. Soc.* **143**, 4433 (2021).

[12] Flechtner, K., Kretschmann, A., Steinrück, H.-P. & Gottfried, J. M. NO-induced reversible switching of the electronic Interaction between a porphyrin-coordinated cobalt ion and a silver surface. *J. Am. Chem. Soc.* **129**, 12110 (2007).

[13] Knecht, P., Reichert, J., Deimel, P. S., Feulner, P., Haag, F., Allegretti, F., Lee, T.-L., Garnica, M., Schwarz, M., Auwärter, W., Ryan, P. T. P., Duncan, D. A., Seitsonen, A. P., Barth, J. V. & Papageorgiou, A. C. Conformational control of chemical reactivity for surface-confined Ru-porphyrins. *Angew. Chem. Int. Ed.* **60**, 1656 (2021).

[14] Woodruff, D. P. Adsorbate structure determination using photoelectron diffraction: Methods and applications. *Surf. Sci. Rep.* **62**, 1 (2007).

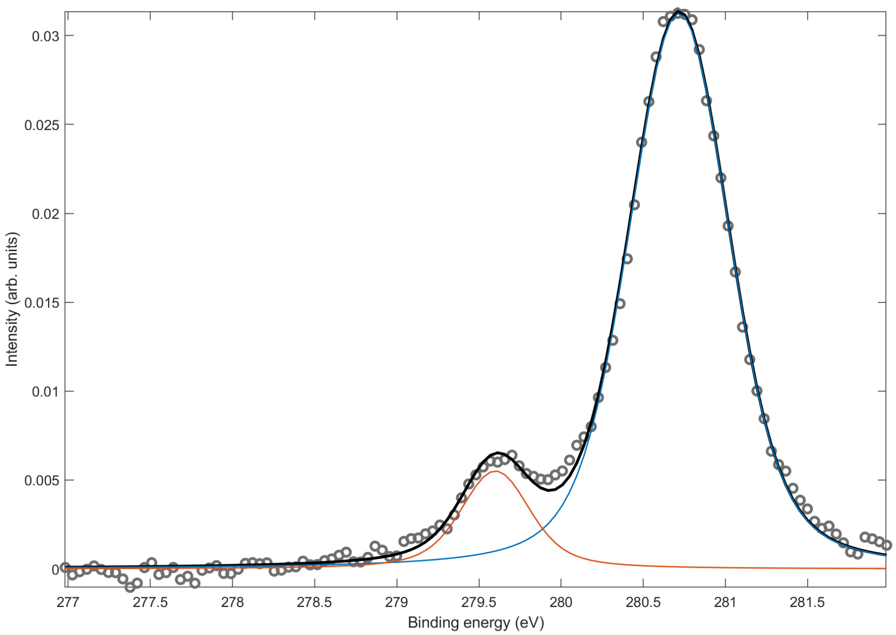

Figure S2: Fitted spectra of the Ru $3d_{5/2}$ signal resulting from the sum of all spectra used for the NIXSW analysis. Two different components can be clearly distinguished, Ru-TPP (orange fit) and Ru(IMe)-TPP (blue fit). The fitting parameters derived from this analysis were used for the analysis of spectra recorded at different photon energies, shown in Figure S3.

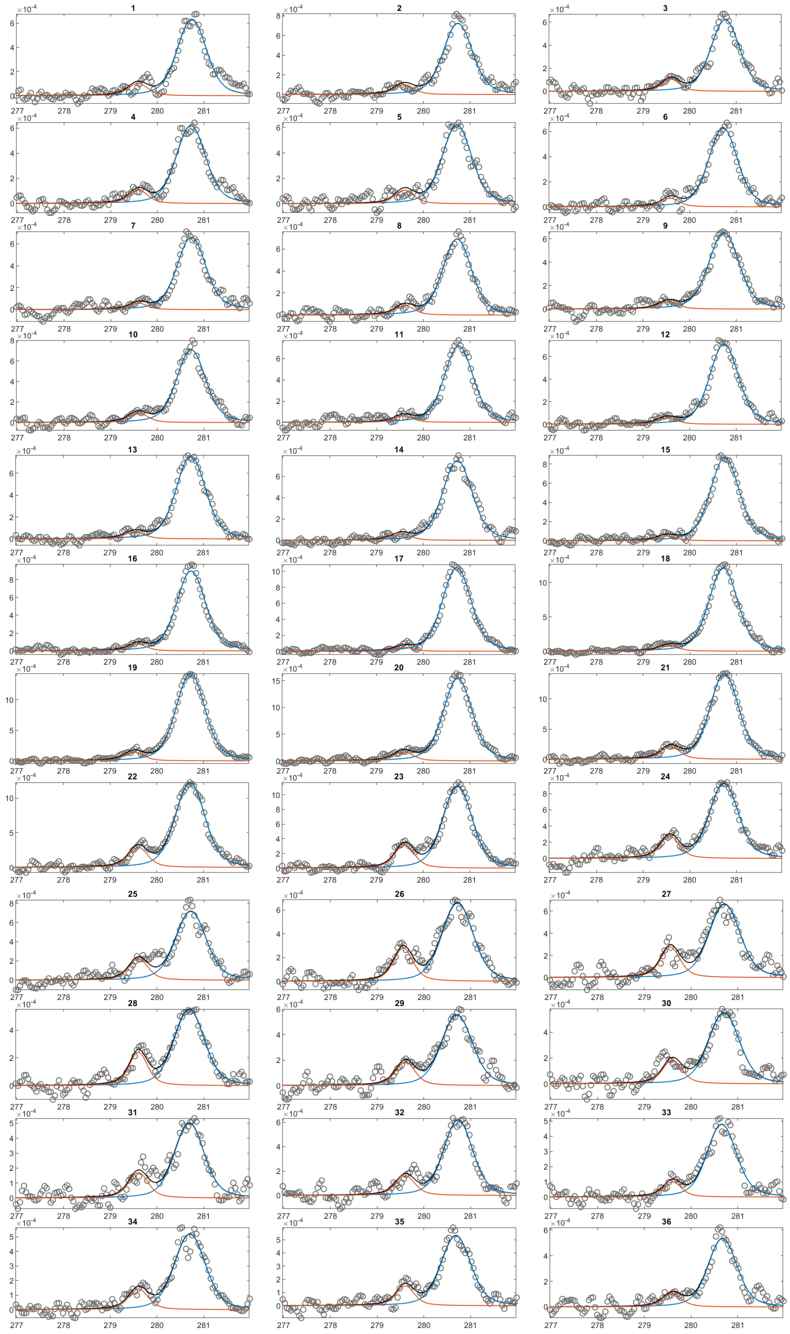

Figure S3: Fits for the Ru $3d_{5/2}$ spectra (intensity in arbitrary units *vs.* binding energy in eV) of the NIXSW analysis presented in the manuscript Figure 1c,d. The two different components correspond to the Ru center in Ru-TPP (orange fit) and Ru(IMe)-TPP (blue fit). The different spectra correspond to different photon excitation energies.

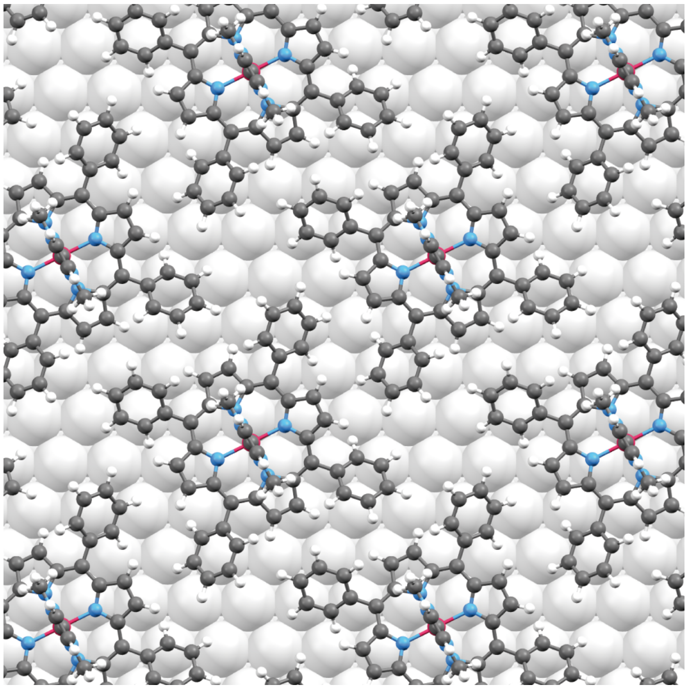

Figure S4: DFT model of Ru(IMe)-TPP on Ag(111). Ru, C, N, H, and Ag atoms are displayed in raspberry, gray, blue, white, and silver, respectively.

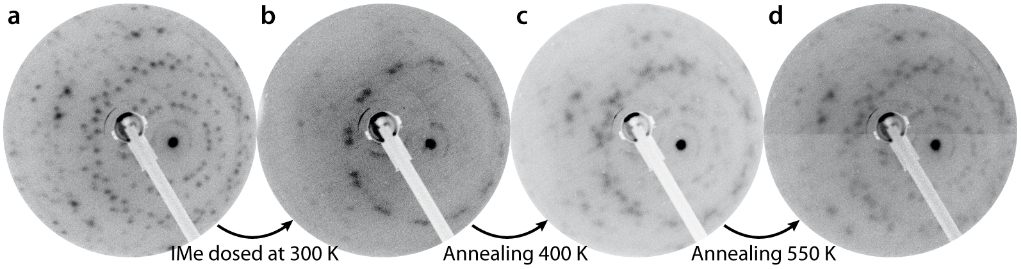

Figure S5: LEED (primary electron energy 20 eV) of Ru-TPP upon ligation on Ag(111): (a) compressed phase Ru-TPP. (b) surface reconstruction upon IMe ligation of Ru-TPP. (c,d) Reversal of the reconstruction upon desorption of IMe by thermal annealing at 400 K / 550 K.

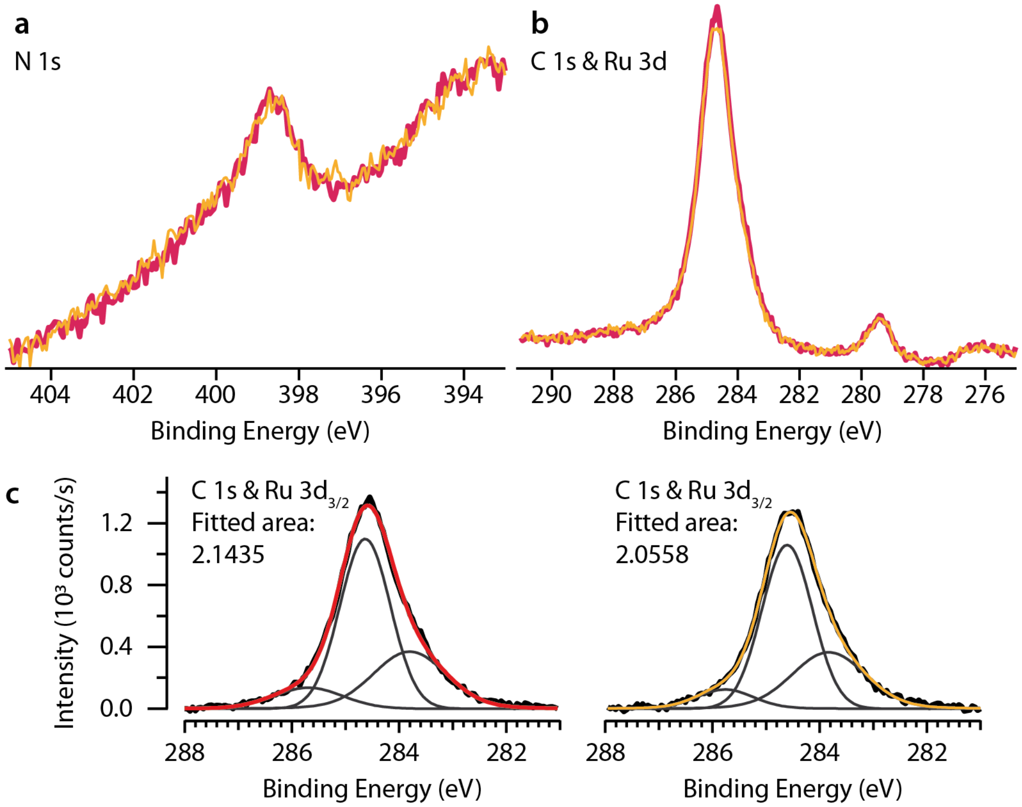

Figure S6: XPS of the (a) N 1s and (b) C 1s & Ru 3d regions corresponding to a freshly prepared compressed phase, single layer coverage of Ru-TPP on Ag(111) (red) and to such a layer after IMe deposition and subsequent annealing to 550 K (orange). The spectra demonstrate the reversible adsorption of IMe, leaving the Ru-TPP layer on Ag(111) unaffected. (c) Quantification of the most intense XPS peaks (area of C 1s & Ru $3d_{3/2}$ peak, left for the spectra before and right for the spectra after) yields a difference of 4%, indicating negligible difference in the Ru-TPP coverage after this process. The data presented by black lines correspond to the raw data after subtraction of Shirley background. These were fitted by Voigt profiles.

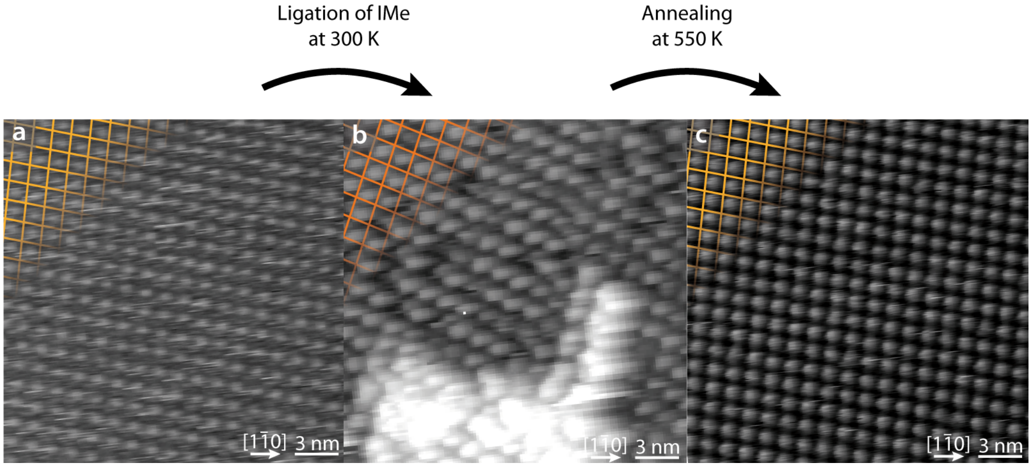

Figure S7: Large scale STM images of the phase transformation upon IMe adsorption. (a) Ru-TPP self-assembled in the compressed phase before IMe ligation (0.6 V, 90 pA, 300 K). (b) Ru(IMe)-TPP self-assembled in the square phase, second layer contributions are visible on the bottom (−2.1 V, 50 pA, 110 K). (c) Ru-TPP in the compressed phase after desorbing IMe ligands (1.3 V, 60 pA, 280 K).

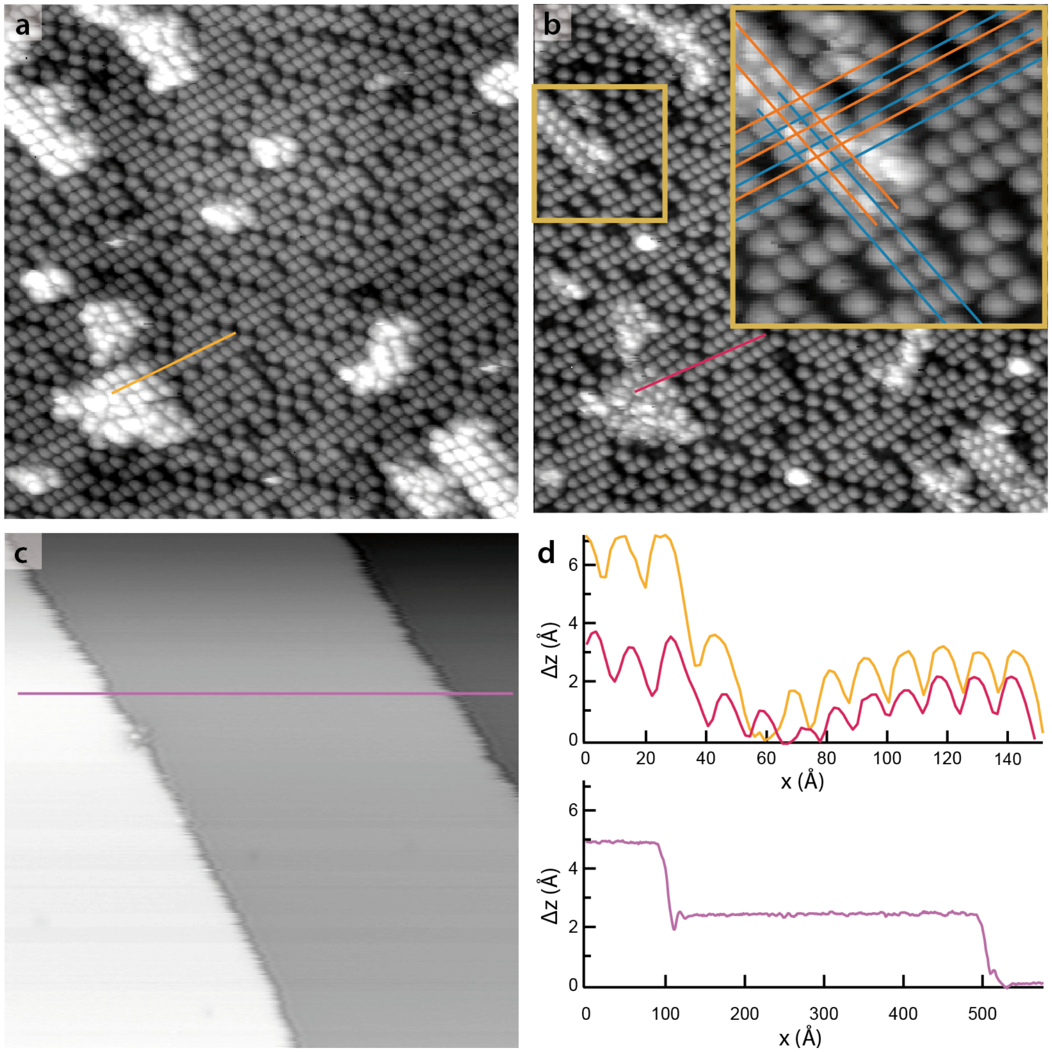

Figure S8: (a,b) Large Scale STM images (a: −2.1 V, 40 pA, 140 K, b: 2.1 V, 60 pA, 140 K) of the IMe porter effect in the system of Ru(IMe)-TPP/Ag(111). (c) STM image (c: 1.3 V, 120 pA, 300 K) of the clean Ag(111) surface displayed for the line profile analysis comparison. (d) Apparent height modulation across the lines indicated with the same colour on the STM images. The apparent height of the molecular adlayers is strongly bias dependent and cannot be correlated directly to the real topographic height. The inset in (b) shows a zoom in with an overlay of the grid of the contact layer (in blue) to the adlayer (in orange): a lateral offset in the stacking of the second layer to the second can be deduced.

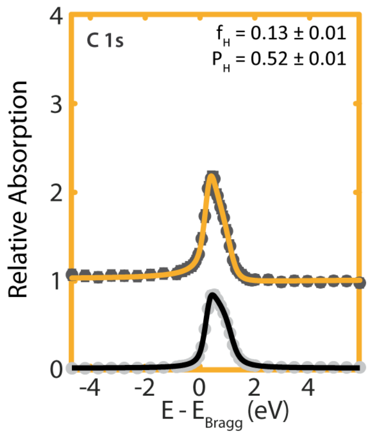

Figure S9: NIXSW results of the C 1s region of the Ru(IMe)-TPP layer. Coherent fraction, f_H, and coherent position, P_H, values are given on the graph.

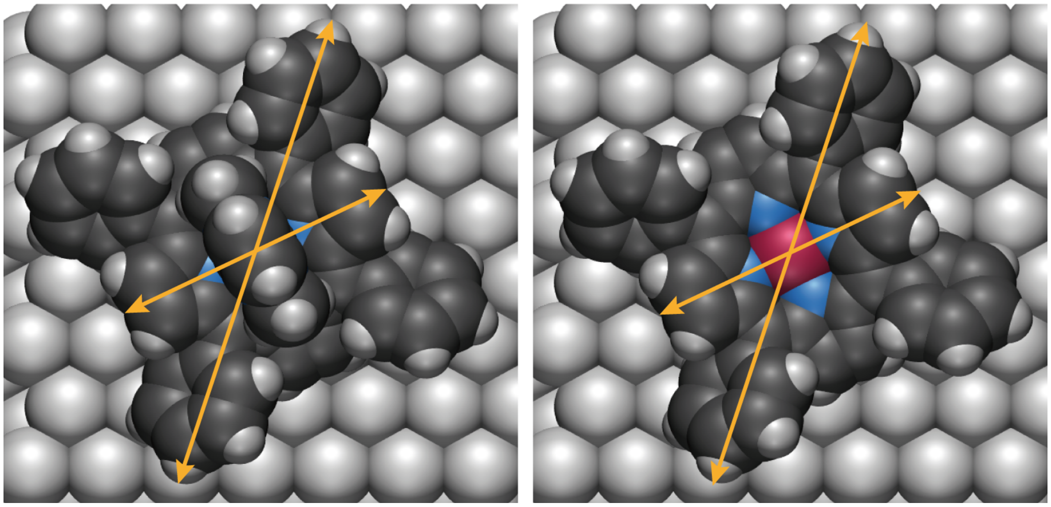

Figure S10: Top view of DFT optimized models of Ru(IMe)-TPP (left, binding energy 3.57 eV) and Ru-TPP (right, binding energy 4.61 eV) on Ag(111). The DFT calculated binding energy of IMe is 1.88 eV, a similar value to the binding energy of the same molecule on the (2×1)-Au(110) (1.98 eV).[15] Ag, C, N, H, and Ru atoms are displayed with their van-der-Waals radii and are indicated by spheres in silver, gray, blue, white, and purple, respectively. The yellow arrows serve as markings for comparison: they have exactly the same dimensions in both images and highlight the similarity of the surface footprint.

[15] Amirjalayer, S., Bakker, A., Freitag, M., Glorius, F. & Fuchs, H. Cooperation of N-heterocyclic carbenes on a gold surface. *Angew. Chem. Int. Ed.* **59**, 21230-21235 (2020).

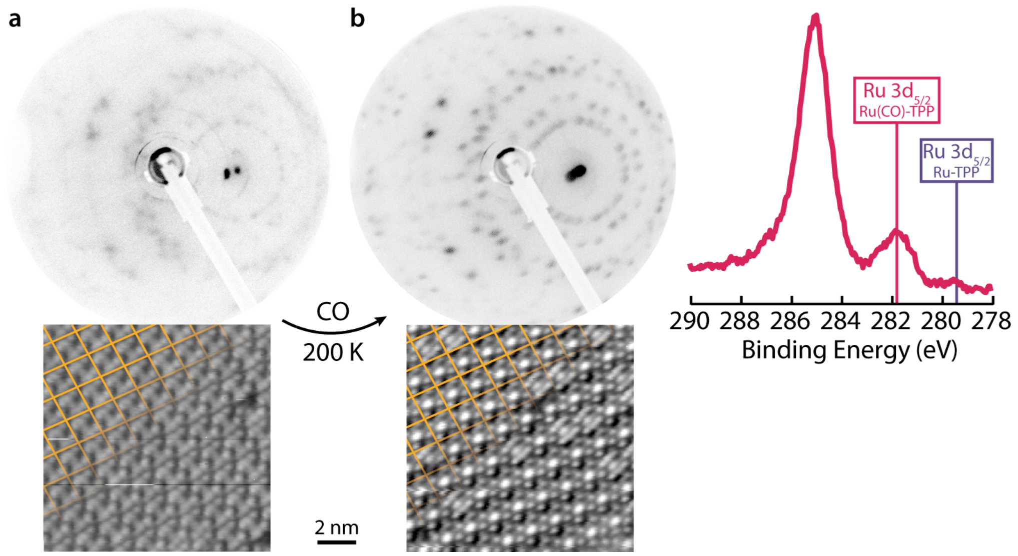

Figure S11: (a) LEED (20 eV) and STM (−1.3 V, 40 pA, 280 K) of compressed phase Ru-TPP on Ag(111). (b) LEED (20 eV), STM (−1.3 V, 30 pA, 210 K) and XPS data of compressed phase Ru-TPP following CO ligation at 200 K. The XPS spectrum and the LEED image were measured on the same surface. The shift in binding energy of the Ru $3d_{5/2}$ peak for the Ru(CO)-TPP (pink marker, 281.8 eV) compared to pristine Ru-TPP (purple marker) shows that CO ligated to the Ru-TPP molecules. Notably, no change in the superstructure periodicity is observed following the CO ligation.

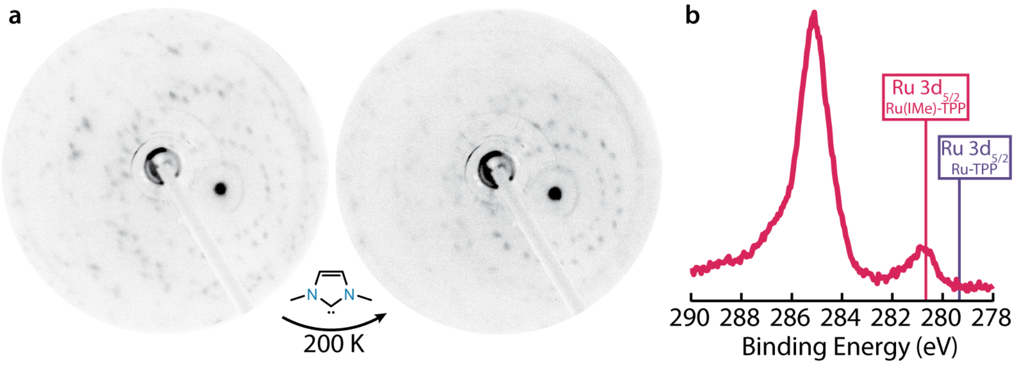

Figure S12: (a) LEED of compressed phase Ru-TPP (left) and of Ru(IMe)-TPP after IMe ligation at 200 K (right). There is no change in the superstructure periodicity observed. (b) XP spectrum of the Ru 3d region for Ru(IMe)-TPP after IMe ligation at 200 K. The shift in binding energy of the Ru $3d_{5/2}$ peak (pink marker) compared to pristine Ru-TPP (purple marker) shows that IMe ligated to the Ru-TPP molecules.

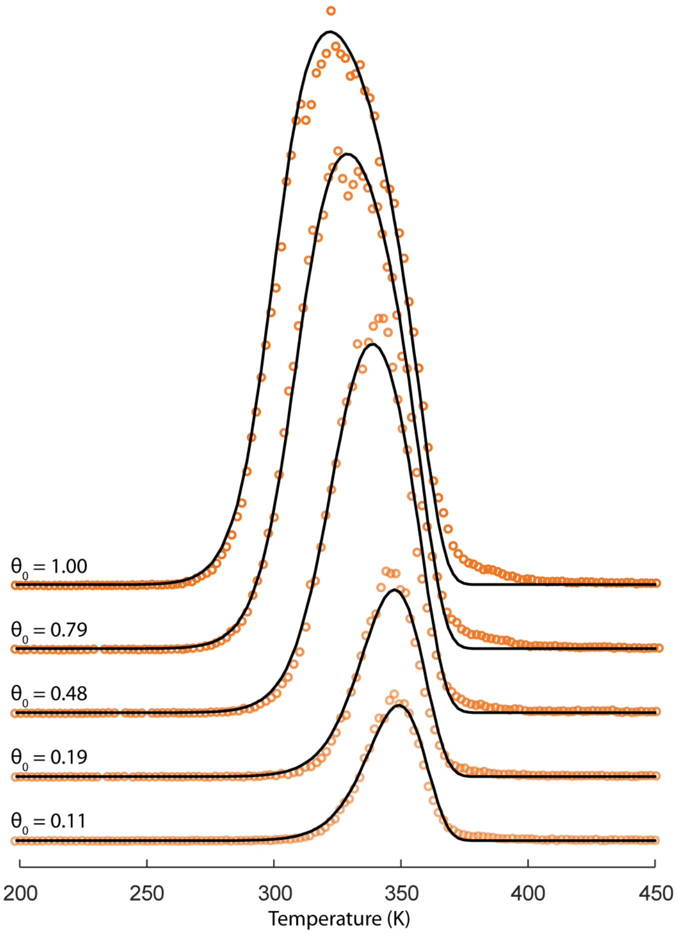

Figure S13: Fitted coverage dependent TPD spectra of IMe (dosed at 200 K) on a compressed single layer of RuTPP on Ag(111). The fitting model includes a coverage-dependent desorption energy of $1.08 - 0.12 \cdot \theta$ eV

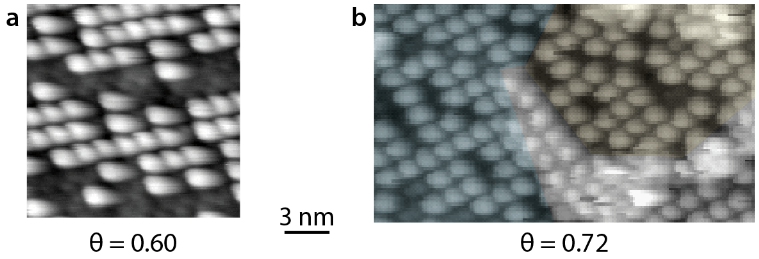

Figure S14: STM investigation of the Ru-TPP structure on Ag(111) as a function of IMe coverage of Ru centers starting with a wetting monolayer of compressed phase Ru-TPP. (a) Up to IMe coverages of $\theta = 0.60$, no structural rearrangement of the porphyrin on the 2D plane is observed. (b) For a coverage $\theta = 0.72$, we find a coexistence of the compressed phase (colored blue) and the square phase (colored yellow) Ru-TPP on Ag(111). In addition, new features corresponding to molecules portered to the second layer (depicted in greyscale) can be identified. STM parameters: a: -1.3 V, 20 pA, 160 K, b: 2.1 V, 60 pA, 140 K.

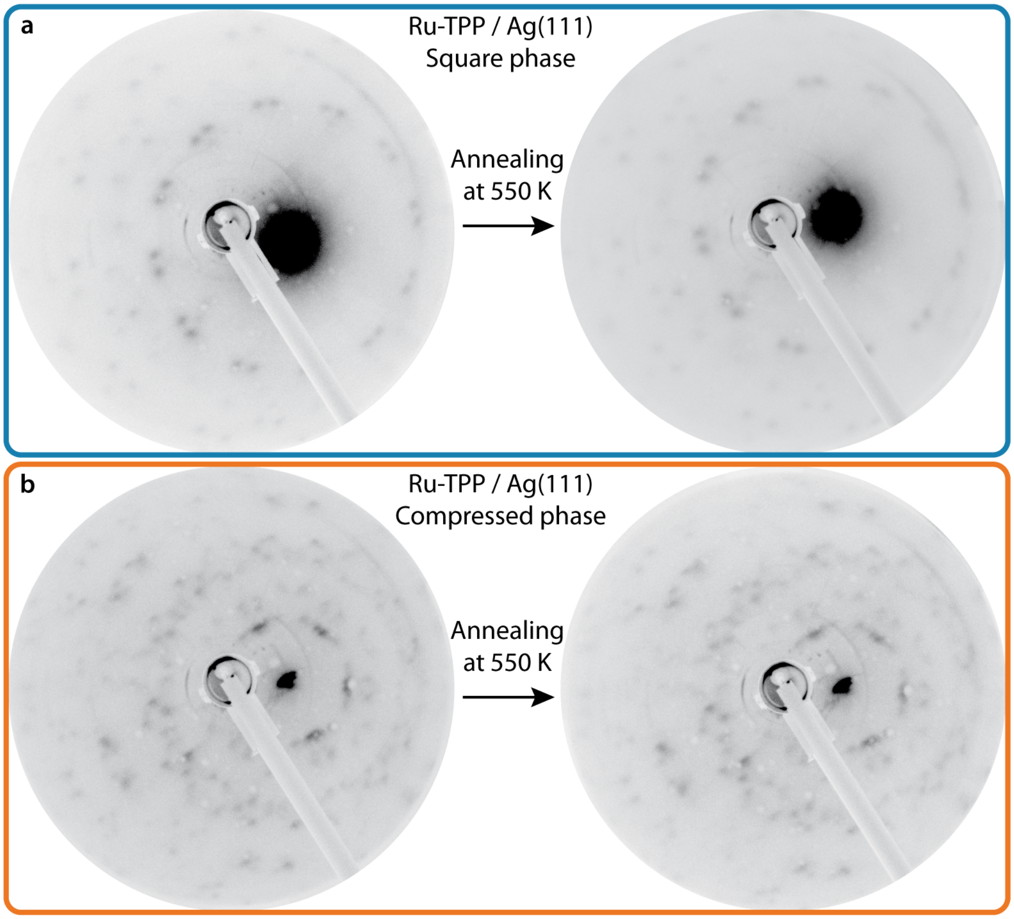

Figure S15: LEED images (primary electron energy 20 eV) of Ru-TPP on Ag(111) in (a) square phase and (b) compressed phase before and after annealing to 550 K. Clearly, the self-assembly is not affected by the annealing process.
